# Methylphenidate and the risk of psychotic disorders and hallucinations in children and adolescents in a large health system

**DOI:** 10.1038/tp.2016.216

**Published:** 2016-11-15

**Authors:** K K C Man, D Coghill, E W Chan, W C Y Lau, C Hollis, E Liddle, T Banaschewski, S McCarthy, A Neubert, K Sayal, P Ip, I C K Wong

**Affiliations:** 1 Department of Pharmacology and Pharmacy, Centre for Safe Medication Practice and Research, Li Ka Shing Faculty of Medicine, The University of Hong Kong, Hong Kong, China; 2 Department of Paediatrics and Adolescent Medicine, Li Ka Shing Faculty of Medicine, The University of Hong Kong, Hong Kong, China; 3 Division of Neuroscience, School of Medicine, University of Dundee, Dundee, UK; 4 Departments of Paediatrics and Psychiatry, Faculty of Medicine, Dentistry and Health Sciences, University of Melbourne, Melbourne, VIC, Australia; 5 CANDAL (Centre for ADHD and Neuro-developmental Disorders across the Lifespan), Institute of Mental Health, Nottingham, UK; 6 Division of Psychiatry and Applied Psychology, School of Medicine, University of Nottingham, Nottingham, UK; 7 Department of Child and Adolescent Psychiatry and Psychotherapy, Central Institute of Mental Health, Medical Faculty Mannheim, Heidelberg University, Mannheim, Germany; 8 School of Pharmacy, University College Cork, Cork, Ireland; 9 Department of Paediatrics and Adolescents Medicine, University Hospital Erlangen, Erlangen, Germany; 10 Centre for Paediatric Pharmacy Research, Research Department of Practice and Policy, UCL School of Pharmacy, London, UK

## Abstract

Previous studies have suggested that risk of psychotic events may be increased in children exposed to methylphenidate (MPH). However, this risk has not been fully examined, and the possibility of confounding factors has not been excluded. Patients aged 6–19 years who received at least one MPH prescription were identified using Hong Kong population-based electronic medical records on the Clinical Data Analysis and Reporting System (2001–2014). Using the self-controlled case series design, relative incidence of psychotic events was calculated comparing periods when patients were exposed to MPH with non-exposed periods. Of 20,586 patients prescribed MPH, 103 had an incident psychotic event; 72 (69.9%) were male and 31 (30.1%) female. The mean age at commencement of observation was 6.95 years and the mean follow-up per participant was 10.16 years. On average, each participant was exposed to MPH for 2.17 years. The overall incidence of psychotic events during the MPH exposure period was 6.14 per 10,000 patient-years. No increased risk was found during MPH-exposed compared with non-exposed periods (incidence rate ratio (IRR) 1.02 (0.53–1.97)). However, an increased risk was found during the pre-exposure period (IRR 4.64 (2.17–9.92)). Results were consistent across all sensitivity analyses. This study does not support the hypothesis that MPH increases risk of incident psychotic events. It does indicate an increased risk of psychotic events before the first prescription of MPH, which may be because of an association between psychotic events and the behavioural and attentional symptoms that led to psychiatric assessment and initiation of MPH treatment.

## Introduction

Attention deficit/hyperactivity disorder (ADHD) is a common neurodevelopmental disorder characterised by a persistent pattern of inattention, hyperactivity and impulsivity that is pervasive across different settings.^1^ It is common among school-aged children and adolescents with a worldwide prevalence around 5%.^2^ ADHD has a diverse range of adverse outcomes in health, intellectual and psychosocial well-being.^3^ Children with ADHD frequently experience low academic attainment, rejection by peers and low self-esteem.^4^ In addition, ADHD is frequently comorbid with other mental health problems such as conduct disorders and substance misuse.^5, 6, 7^ Therefore, behavioural intervention and/or drug treatment is usually required to mitigate these symptoms and impairments. The guidelines from the National Institute for Health and Clinical Excellence (NICE) in the United Kingdom recommend the use of methylphenidate (MPH), dexamfetamine and atomoxetine when drug intervention is considered appropriate for the management of ADHD.^8^ In the past two decades, ADHD treatment prescribing trends have risen rapidly in the United States (US),^9^ Canada,^10^ the UK,^11, 12^ Germany^13^ and Hong Kong (HK).^14^ As suggested by a very recently published commentary and a meta-analysis, it is important to evaluate the risks (that is, adverse effects) as well as benefits of MPH treatment in clinical practice.^15, 16^

MPH is the most commonly prescribed medication treatment for ADHD.^11, 14^ It acts primarily as a dopamine–norepinephrine reuptake inhibitor by binding to and blocking dopamine transporters.^17^ As increased levels of synaptic dopamine are implicated in the generation of psychotic symptoms,^18^ the pharmacological mechanism of MPH also implies a potential to induce psychotic symptoms and disorders.^19^ Data from the UK Medicines and Healthcare Products Agency's Yellow Card scheme^20^ showed that out of 1335 adverse drug reaction (ADR) reports for MPH received by the end of July 2015, 663 were associated with psychiatric disorders, making these the most frequently reported class of ADR. Among these reports, 105 cases (15.8%) reported hallucinations, psychosis or psychotic disorders, or schizoaffective disorders. Mosholder *et al.*^21^ reviewed and analysed data from the medication manufacturers on ADHD drugs from 49 randomised controlled clinical trials. They identified a total of 11 psychosis/mania adverse events during 743 person-years of follow-up in 5717 individuals (1.48 events per 100 person-years or one event in every 70 years of treatment) compared with none reported with placebo, giving a number needed to harm of 526.

These reports raise the possibility that MPH treatment may be associated with an increased risk of psychosis or related events. In 2007, the European Commission requested a referral to the Committee for Medicinal Products for Human Use under Article 31 of Directive 2001/83/EC, as amended, for MPH because of safety concerns.^22, 23^ One of the main potential safety areas that was evaluated concerned adverse psychiatric events including psychosis. In 2009, the Committee for Medicinal Products for Human Use concluded that the benefit-risk of MPH remains favourable in the authorised indication, but that further research on safety is needed.^23^ In view of the dopamine agonist effect of MPH, the observed reports of an association between MPH and hallucinations, psychosis or psychotic disorders (collectively described here as 'psychotic events')^17^ and increasing use of MPH worldwide, there is a clear need for a systematic investigation into the association between MPH and psychotic events. One recent study has investigated the relationship between ADHD and schizophrenia spectrum disorders.^24^ Although the authors found an increased risk of psychotic disorder in MPH users, they recognised that several important pieces of potentially confounding clinical information were not available to the study team. These included the following: the severity of ADHD symptoms; family history of psychosis; and history of substance misuse. Thus, it is not possible to determine whether the finding of a positive association between MPH treatment and psychosis in this analysis was in fact a consequence of confounding by other important differences between MPH-treated patients and the control group that themselves increase the likelihood of receiving MPH treatment.^24^

The aim of this study was to address these shortcomings by examining the association between MPH and the risk of psychotic events using the self-controlled case series (SCCS) method. With this method, cases act as their own controls and observations are made within cases over time. In this study, comparisons were made within a clinically referred sample of individuals with psychotic events who have been prescribed MPH in the setting of a territory-wide hospital database in HK. We hypothesised that MPH treatment will be associated with increased incidence of psychotic events following MPH exposure.

## Materials and methods

### The Clinical Data Analysis And Reporting System

This study was conducted using the Clinical Data Analysis and Reporting System (CDARS), an electronic health record database developed by the Hong Kong Hospital Authority (HA), a statutory body that manages all public hospitals and their ambulatory clinics in HK. The service is available to all HK residents (over 7 million) and covers ~80% of all hospital admissions in HK.^25^ Data from CDARS have proved to be reliable for use in research and have been used for various pharmacoepidemiological studies.^25, 26, 27, 28, 29^ These have included MPH investigation of prescribing trends in HK, and of the association between MPH treatment and risk of trauma.^14, 30^ CDARS has also been used to investigate psychiatric and neurological ADRs.^31, 32^ Patient-specific clinical data in CDARS include diagnosis, prescription, and information on admission and discharge, all of which are recorded by trained clinicians. Other patient-specific data such as demographics, payment method, prescription and pharmacy-dispensing information are entered by other trained staff.^33^ CDARS contains the records of all in-patient, out-patient and emergency room admissions in HA clinics and hospitals since 1995. Records are anonymised to protect patient confidentiality. Unique patient reference numbers are generated to facilitate data retrieval. Detailed descriptions of CDARS can be found elsewhere.^14, 34^ Previous validation studies have shown high positive predictive values for various medical diagnoses.^25, 28^

### SCCS design

The SCCS^35^ study design was specifically selected to investigate the association between MPH and psychosis. This method has been used previously to investigate the effects of MPH on emergency room admission in HK.^30^ In SCCS, each patient serves as their own control and the modelling is conditional in that all cases will have experienced the outcome of interest at some stage during the study window.^36^ This study design relies on within-person comparisons in a population of individuals who have experienced both the outcome and exposure of interest.^36^ Incidence rate ratios (IRRs) are derived by comparing the rate of events during exposed periods (on medication) with the rate during all other observed time periods (off medication). A major advantage of this design is that the potential time-invariant confounding effect of fixed characteristics (both recorded and unrecorded) that vary between individuals and may underlie disease severity, such as genetic and socioeconomic factors, are removed. The underlying risks of psychotic events among MPH users and non-users are likely to be different because of factors relating to ADHD and its comorbidities, and are difficult to control for in most observational study designs. This can be a major issue in cohort or case–control studies as the comparison group may not be appropriately selected. The SCCS design, in which confounding between individuals is controlled implicitly, is able to address these factors that may not be controlled in classic epidemiological study designs. Furthermore, we are able to adjust for factors that vary with time, particularly age and seasonal effects, as both the MPH treatment prescribing and psychiatry visits have age and seasonal patterns.^37, 38^

### Case identification

Individuals aged 6–19 years who received at least one prescription of MPH with at least one psychotic disorder and/or hallucination diagnostic code (psychotic events) during the study period (January 2001 to December 2014) were identified in CDARS. The psychotic event codes were identified through the diagnostic codes from the International Classification of Diseases, Ninth Revision, Clinical Modification (ICD-9-CM). Patients with psychotic events before the start of follow-up were excluded. The included diagnosis codes are listed in Supplementary Table 1. Only MPH and atomoxetine are licensed for the treatment of ADHD in HK; therefore, the observation periods were censored by atomoxetine treatment to avoid a co-prescribing situation, which may affect comparisons. In HK, ~5% of treated ADHD patients received both MPH and atomoxetine.^14^ Individual observation periods commenced on January 2001, or the sixth birthday of the patient (whichever was later), and ended on December 2014, the twentieth birthday of the patient, date of receiving atomoxetine treatment or the date of registered death (whichever was earlier). We commenced follow-up at 6 years of age because MPH is not recommended for younger children.^39^ As the aim of this pharmacovigilance study is to investigate the association between MPH and incident psychotic events, a diagnosis of ADHD was not an inclusion criterion.

### Exposures and outcomes

**Figure 1:**
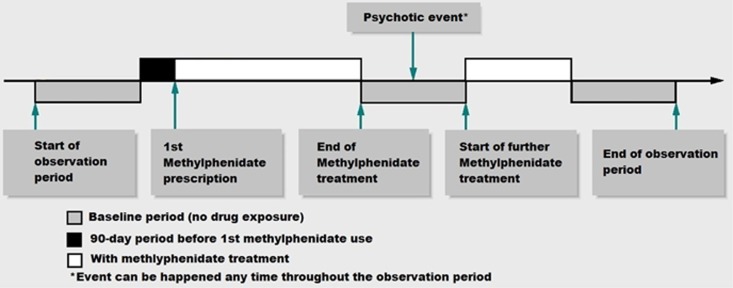
Illustration of the self-controlled case series study design.

For each included participant, records of all MPH prescriptions and psychotic events were identified. All formulations of MPH (standard and extended release) and all strengths were included in the analysis. ‘Exposed periods' were defined as time on-medication and were estimated by the duration between prescription start and end date recorded in CDARS for each prescription. Over 99% of prescriptions have the intended start and end date of the treatment recorded in our data set. Daily dosages and quantity prescribed were used to determine the duration of treatment if prescription end date was not available. The median values for exposure duration were imputed when the above information was missing. Periods within the observation period other than exposure periods were classified as baseline periods. We did not assume that participants received continuous treatment upon initiation of MPH. This is because clinicians may offer drug holidays to ADHD patients during school holidays, and treatment may be stopped and started for various reasons. A pictorial presentation of the study design and timeline for a single hypothetical participant is given in Figure 1. Psychotic events were identified through ICD-9-CM codes (Supplementary Table 1) by CH and KS. The corresponding date of a psychotic event was identified by the event date and only the incident psychotic event for each patient was included in the analysis. We conducted a validation analysis by reviewing the information in CDARS. Through this we identified that in 98 out of 103 (95.1%) cases the diagnosis of a psychotic event was confirmed by a hospital paediatrician and/or psychiatrist. All included patients were under the care of specialist clinics managing childhood mental health conditions. Consequently, the risk of misdiagnosis is considered to be low.

### Statistical analysis

The primary analysis investigated the relationship between MPH treatment and the occurrence of incident psychotic events. This was calculated by comparing the rate of psychotic events during exposed periods to that during baseline periods. Adjusted IRR and the corresponding 95% confidence intervals (CIs) were calculated using conditional Poisson regression, adjusting for age, in 1-year bands, and season. As the psychotic event itself may potentially have an impact on the likelihood of receiving MPH treatment, which in turn may introduce bias into the risk estimate during treatment, a 90-day pre-exposure period was added to remove the short-term impact of this effect (Figure 1). For a psychotic event that occurred on day 1 of MPH treatment, we reviewed the temporal relationship of this event and treatment, that is, whether it was before or after initiation of MPH. If an event occurred before the MPH treatment was prescribed, it would be classified as pre-exposure period instead of day 1. A significance level of 5% was used in all statistical analyses. Microsoft Excel and Statistical Analysis System (SAS) v9.3 (SAS Institute, Cary, NC, USA) were used for data manipulation and analysis.

### Code availability

Analysis codes are available upon request to the corresponding author.

### Sample size calculation

Using the approach and equation suggested by Musonda *et al.*,^40^ an IRR of 2 with 80% power (two-sided 95% CI) could be detected with a minimum of 76 psychosis cases.

### Sensitivity analyses

Several sensitivity analyses were planned to test the validity and robustness of the initial study results: (1) Alternative analyses were conducted based on different drug non-adherence scenarios. Each exposed period was further extended by adding 1–10 weeks after the end of an exposed period to assess this effect. (2) To assess the sensitivity of age-banding used, an analysis with a 6-month age band rather than annual bands was conducted. (3) Additional analyses were conducted on a subset of patients with more than 10 weeks of MPH exposure in order to test the effects of more prolonged medication exposure. (4) Patients with a diagnosis of substance misuse/dependence (ICD-9-CM: 303-305) were removed from the analysis. (5) The individual observation period was censored by the date of prescription of any antidepressant or antipsychotic medications during the study period. (6) The outcome was restricted to ICD-9-CM psychotic disorders only meaning that those with hallucinations (ICD-9-CM: 780.1) were removed from the analysis. (7) Cases where the event occurred on the first day of prescription were removed. (8) Different washout periods (7–21 days) were implemented before the initiation of MPH treatment and these periods were excluded from the analysis. (9) The observation period was started at January 2001, the sixth birthday of the patient, the first observed date of ADHD diagnosis or the first date of MPH treatment, whichever occurred later. (10) Different lengths of pre-exposure period (30 and 60 days) were used.

### Ethical approval

This study protocol was approved by the Institutional Review Board of the University of Hong Kong/Hospital Authority Hong Kong West Cluster (Reference Number: UW12-136).

## Results

**Figure 2:**
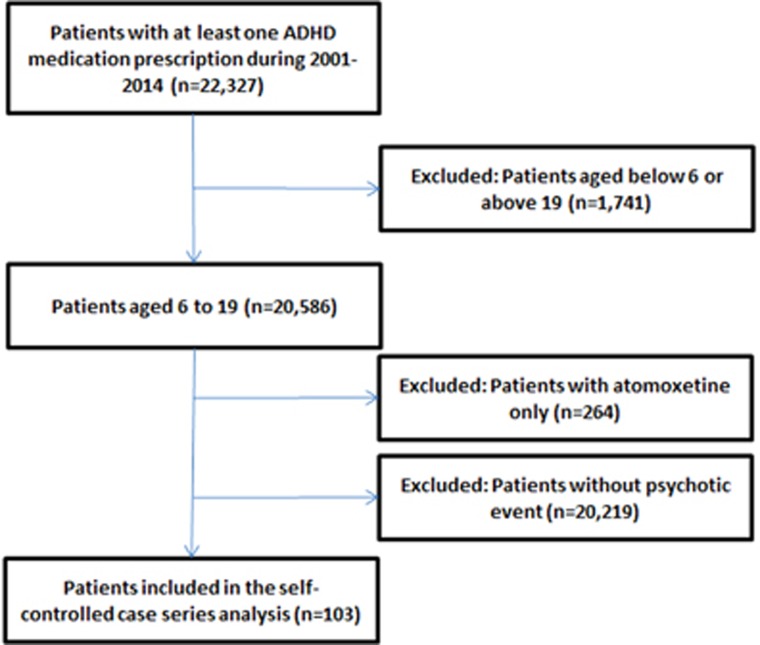
Flowchart of patient identification. ADHD, attention deficit/hyperactivity disorder.

Among 20,586 patients with MPH prescriptions, 103 were included in the primary analysis (Figure 2), of which 72 (69.9%) were male and 31 (30.1%) were female. The mean age at commencement of observation was 6.95 years and the mean duration of follow-up per participant was 10.16 years. The mean exposure to MPH was 2.17 years per participant. The median length of each prescription was 70 days. In all, 76 out of 103 patients had a clinical ADHD diagnosis and the median age of diagnosis was 9.5 years (interquartile range 8.2–11.7). There were 103 incident psychotic events, of which 78 occurred during baseline periods and 25 occurred during the MPH treatment period (Table 1). Among the 103 cases, 80 were psychosis cases (ICD-9-CM: 298.0, 298.1, 298.3, 298.8, 298.9), 20 were hallucinations (ICD-9-CM: 780.1) and 3 were other psychotic disorders (other codes in Supplementary Table 1). The overall incidence of psychotic events during the MPH treatment period was 6.14 per 10,000 patient-years. No participants died during the study period. Broader psychiatric comorbidities for these patients are shown in Table 2.

The primary analysis indicated no statistically significant association between MPH treatment and occurrence of incident psychotic events (Table 3). After adjusting for age and season, the IRR was 0.98 (95% CI 0.52–1.86). After including a 90-day pre-exposure period, a similar result was found with an IRR during treatment of 1.02 (95% CI 0.53–1.97). Compared with the baseline, an IRR of 4.64 (95% CI 2.17–9.92) was found in the 90-day pre-MPH treatment period (Table 3). Direct comparison between the risk of psychotic events during the MPH treatment period and the pre-exposure period showed that the corresponding risk during MPH treatment period is lower than during the pre-exposure period (IRR=0.13; 95% CI 0.04–0.50; *P*-value 0.003). The additional sensitivity analyses all gave similar results (Table 3 and Supplementary Figure 1).

## Discussion

These data do not support the presence of an association between the use of MPH and the development of incident psychotic events (IRR=1.02 (95% CI 0.53–1.97)). However, a positive IRR was observed in the pre-MPH treatment periods (IRR=4.64, 95% CI 2.17–9.92), which was markedly elevated relative to the risk of incident psychotic events during MPH treatment.

Possible reasons for an increased risk of incident psychotic events before starting MPH treatment include the co-occurrence of transient psychotic events with ADHD, or with clinical contact and observation in the period leading up to initiation of MPH. It is well recognised that patients with ADHD are prone to cognitive, emotional and behavioural comorbidities.^7^ These comorbidities may increase the likelihood of psychiatric consultation, which may consequently increase both the chance of incident psychotic events being identified and being prescribed MPH. This increased diagnosis of incident psychotic events in the period before the first ever MPH treatment may also explain the ADR reports of psychiatric adverse events for MPH, as cited in the literature.^41, 42, 43, 44, 45^ If, as our evidence suggests, the diagnosis of incident psychotic events is higher before the MPH treatment, this may increase the likelihood of subsequent diagnosis of recurrent psychotic events, and thus reports of psychotic events associated with MPH treatment in clinical practice.

Several case reports of MPH-induced psychosis-like symptoms in children have been published.^41, 42, 44, 45^ However, most of these patients also had other psychiatric conditions such as emotional and behavioural disorders. Therefore, it is important to note that these reported psychotic events may not be induced by ADHD medication but could simply reflect the deterioration of a coexisting psychiatric disorder. In addition, the important finding reported here of an increased risk of psychotic events' pretreatment may not be observed in a classic cohort study, where patients with either events or exposures before the commencement of study are usually excluded.

Mosholder *et al.*^21^ reviewed data on hallucinations and other psychotic symptoms associated with the use of ADHD drugs (included MPH, modafinil, dextromethylphenidate, amphetamine and atomoxetine) from 49 randomised controlled clinical trials. Although the aggregated adverse-event data found that a rate of psychosis/mania event was 1.48 per 100 person-years in ADHD treatment group, only four events were reported in trials for MPH products; all were from transdermal patch treatment only and none for oral MPH products. In HK, only oral MPH products are available.

A recent study in Canada recruited a group of parents with severe mental illness and used questionnaires to investigate the relationship between stimulant medication use and psychotic symptoms.^43^ The authors identified 24 patients who had been exposed to stimulants in lifetime. Among them, 15 had lifetime occurrence of psychotic and related symptoms with adjusted odds ratio of 4.41 when comparing with individuals who had never used stimulants. This study included a group of high-risk individuals with psychiatric family history which, as stated by the authors, were unable to test whether family history moderates the risk of psychotic symptoms.^43^ In addition, the temporal relationship of psychotic symptoms identified and reported use of stimulants were not known in this study. Thus, the findings only showed an association but do not prove causality. We found an IRR of 4.64 before the initiation of MPH treatment that was comparable to the odds ratio in this Canadian study. Therefore, our study results raised the possibility of reverse causality between MPH use and psychotic disorders.

Another recent cohort study in Taiwan^24^ investigated the association between MPH and schizophrenia spectrum disorders, and concluded that there was an increased risk of psychotic disorder in ADHD patients taking MPH compared with non-users (adjusted hazard ratio=1.20). However, our data find a lower and not statistically different estimate (IRR=1.02). The discrepancy between the results may be explained by differences in study design. We applied the SCCS design, which controls for the effects of unmeasured confounders more robustly, as the within-person study design controls implicitly for confounders that do not vary over time.^36^ In the Taiwanese study, as acknowledged by the authors, several potential confounders remained unmeasured. These included the baseline severity of ADHD symptoms, and family history of psychosis, both of which may affect the results of a cohort study. These potential confounders, even though they were not captured in our database, should not have an impact on the findings reported here using the SCCS design. This may explain why our study resulted in an estimate closer to 1. Although the SCCS design was able to control confounders that do not vary over time, it may not be able to control for changes in ADHD severity. ADHD severity may change over time, which is likely to be associated with MPH treatment and is potentially a risk factor for psychotic symptoms. This bias would likely result in an overestimation of the true risk of association, as patients with more severe ADHD are more likely to be treated as well as being at higher risk for psychotic events. Therefore, even if this was the case, it is unlikely to change the direction of our conclusions.

There are a number of limitations in our study. First, CDARS does not have linkage to data from private medical practitioners. Therefore, we were not able to include prescriptions from the private sector, which may potentially lead to exposure misclassification. However, in HK, the public sector is the main provider of specialist care^46^ and there are very few private child and adolescent psychiatrists.^14, 47^ As a consequence, patients who require long-term care, such as those with neurodevelopmental disorders and ADHD, are generally treated in the public health-care sector,^14^ and the vast majority should have been included in this study. In addition, our cohort only included clinically referred patients who had sufficiently severe ADHD symptoms and/or impairment to be prescribed MPH treatment. Therefore, our cohort may have a higher baseline risk than non-medicated patients. However, as the aim of the study was to evaluate the effect of MPH on the risk of a psychotic event, our cohort included all patients with MPH treatment within the public health-care system. Hence, our cohort is highly representative. Furthermore, as we applied a SCCS design, individual baseline risk will not affect our study results and conclusion. Second, similar to other pharmacoepidemiological studies using automated databases, CDARS provides the data on drug prescription, but not on adherence (compliance) to medication, and this may lead to misclassification of exposure periods. However, these potential limitations due to non-compliance with medication were addressed to some extent in the sensitivity analysis, and the results remained similar. Third, despite having identified that in 95.1% cases, the diagnoses of a psychotic event was confirmed by a hospital paediatrician and/or a psychiatrist, we cannot rule out the possibility of under-diagnosis in which the sensitivity of diagnosis may depend on treatment status. For example, physician visits may be more frequent during periods with MPH treatment, and therefore diagnosis of psychotic events/hallucinations more likely to come to clinical attention and diagnosis. This differential under-diagnosis may potentially lead to an overestimation in the IRR during treatment. However, this again would be unlikely to affect the study conclusions. Fourth, the upper limit of CI for the reported IRR during MPH treatment is just below 2; we cannot exclude the possibility of the risk of incident psychotic events doubling during treatment as we do not have sufficient statistical power to detect an IRR below 2. However, even if there is an increased risk, the absolute increased risk would be small, as the absolute risk of incident psychotic events was 1 per 1629 patient-years. More importantly, the IRR is statistically significantly lower during MPH treatment than for the pre-treatment period; therefore, there is no evidence to support an increased risk associated with the MPH treatment. Fifth, we cannot evaluate the deterioration, persistence or recurrence of the psychotic disorders after the incident diagnosis date. In particular, for patients with schizophrenia, we are unable to determine the risk of deterioration, owing to insufficient sample size. Further study should be conducted to evaluate the risk of deterioration, persistence or recurrence of psychotic symptoms in patients with a previous history of psychotic events before the initiation of the MPH treatment. Sixth, as we had a comparatively long follow-up time, other time-varying confounding factors may affect our study results. We hypothesised that the use of other psychiatric medications may affect the association between MPH and psychotic events. Therefore, we conducted a sensitivity analysis by censoring patient-time after the first antidepressant or antipsychotic prescription, and the results were consistent with our initial findings. Lastly, although there is no evidence to suggest that Chinese patients respond differently to MPH than other populations, we cannot fully exclude this as a possibility.

## Conclusions

This study does not support the hypothesis that MPH increases risk of incident psychotic events. It does, however, indicate an increased risk of such events before the first prescription of MPH, which may be due to the association between psychotic events and the behavioural and attention symptoms that led to psychiatric consultations and initiation of treatment with MPH.

## Figures and Tables

**Table 1:** Patient characteristics

	*No. of patients*	*%*	*Mean age at baseline (years)*	*s.d.*	*Median daily dosage (mg)*	*IQR* ^*b*^ *of daily dosage (mg)*	*Mean age at event*	*s.d.*	*Median length of prescription (days)*	*Exposed period*		*Unexposed period*	
										*No. of events*	*Total follow-up time (patient-years)*	*No. of events*	*Total follow-up time (patient-years)*
All	103	100	6.95	1.78	20	20	13.5	3.6	70	25	223.0	78	823.6
Male	72	69.9	6.97	1.78	20	20	13.7	3.7	70	15	162.7	57	582.3
Female	31	30.1	6.90	1.81	20	15	13.1	3.4	70	10	60.3	21	241.3

Abbreviation: IQR, interquartile range.

**Table 2:** Psychiatric comorbidities of patients with psychotic events

	*ICD-9-CM*	*Number of patients*	*%*
Acute reaction to stress	308	22	21.4
Adjustment disorder	309	12	11.7
Anxiety disorder	293.84, 300	11	10.7
Autism spectrum disorder	299	22	21.4
Disturbance of conduct not elsewhere classified	312	25	24.3
Specific delays in development	315	16	15.5
Other psychiatric comorbidities^a^	290–319	73	70.9

Abbreviation: ICD-9-CM, International Classification of Diseases, Ninth Revision, Clinical Modification.

a Other psychiatric comorbidities included all other disorders from ICD-9-CM code 290–319 that were not psychosis or listed above.

**Table 3:** Results from the self-controlled case series analyses

	*IRR*	*95% CI*	P-*value*
*Incident psychotic episode (n=103)*			
Period with MPH treatment	0.98	0.52–1.86	0.95
			
*Pre-risk period included*			
90 Days before first MPH treatment	4.64	2.17–9.92	<0.01
Period with MPH treatment	1.02	0.53–1.97	0.95
			
*Sensitivity analyses*			
6-Month age band (*n*=103)			
90 Days before first MPH treatment	3.91	1.85–8.28	<0.01
Period with MPH treatment	0.84	0.46–1.55	0.58
Patients with >10 weeks' MPH exposure (*n*=82)			
90 Days before first MPH treatment	4.38	1.77–10.87	<0.01
Period with MPH treatment	1.07	0.55–2.08	0.85
Censor by antidepressants/antipsychotics (*n*=102)			
90 Days before first MPH treatment	6.67	2.84–15.66	<0.01
Period with MPH treatment	0.92	0.40–2.13	0.84
Remove patients with substance dependence (*n*=87)			
90 Days before first MPH treatment	5.01	2.32–10.81	<0.01
Period with MPH treatment	0.89	0.44–1.78	0.74
Remove hallucination cases (*n*=83)			
90 Days before first MPH treatment	3.37	1.22–9.32	0.02
Period with MPH treatment	1.11	0.53–2.31	0.79
Remove cases with event on the first day of treatment (*n*=102)			
90 Days before first MPH treatment	4.04	1.82–8.95	<0.01
Period with MPH treatment	0.99	0.51–1.92	0.66
Washout period: 7 days before the first treatment (*n*=90)			
90 Days before first MPH treatment	3.90	1.69–9.01	<0.01
Period with MPH treatment	1.01	0.53–1.94	0.97
Washout period: 14 days before the first treatment (*n*=90)			
90 Days before first MPH treatment	4.26	1.84–9.86	<0.01
Period with MPH treatment	1.01	0.53–1.94	0.98
Washout period: 21 days before the first treatment (*n*=89)			
90 Days before first MPH treatment	3.96	1.62–9.69	<0.01
Period with MPH treatment	1.01	0.53–1.95	0.97
			
Start of observation at 1 January 2001, the sixth birthday of the patient, the first observed date of ADHD diagnosis or the first date of MPH treatment, whichever occurred last (*n*=79)			
90 Days before first MPH treatment	3.59	1.13–11.4	<0.01
Period with MPH treatment	1.26	0.61–2.59	0.53
* *60-Day pre-exposure period			
60 Days before first MPH treatment	5.99	2.71–13.22	<0.01
Period with MPH treatment	1.01	0.53–1.95	0.97
30-Day pre-exposure period			
30 Days before first MPH treatment	5.21	1.83–14.81	<0.01
Period with MPH treatment	0.94	0.49–1.81	0.86

Abbreviations: 95% CI, 95% lower confidence interval of IRR; ADHD, attention deficit/hyperactivity disorder; IRR, adjusted incidence rate ratio; MPH, methylphenidate.

## References

[bib1] American Psychiatric AssociationDiagnostic and Statistical Manual of Mental Disorders, 5th edn. American Psychiatric Publishing: Arlington, VA, 2013.

[bib2] Polanczyk G, de Lima MS, Horta BL, Biederman J, Rohde LA. The worldwide prevalence of ADHD: a systematic review and metaregression analysis. Am J Psychiatry 2007; 164: 942–948. 17541055 10.1176/ajp.2007.164.6.942

[bib3] Biederman J, Faraone SV. Attention-deficit hyperactivity disorder. Lancet 2005; 366: 237–248. 16023516 10.1016/S0140-6736(05)66915-2

[bib4] Harpin VA. The effect of ADHD on the life of an individual, their family, and community from preschool to adult life. Arch Dis Child 2005; 90: I2–I7. 15665153 10.1136/adc.2004.059006 PMC1765272

[bib5] Harstad E, Levy S, Abuse CS. Attention-deficit/hyperactivity disorder and substance abuse. Pediatrics 2014; 134: E293–E301. 24982106 10.1542/peds.2014-0992

[bib6] Connor DF, Doerfler LA, ADHD With Comorbid. Oppositional defiant disorder or conduct disorder discrete or nondistinct disruptive behavior disorders? J Atten Disord 2008; 12: 126–134. 17934178 10.1177/1087054707308486

[bib7] Lambek R, Tannock R, Dalsgaard S, Trillingsgaard A, Damm D, Thomsen PH. Executive dysfunction in school-age children with ADHD. J Atten Disord 2011; 15: 646–655. 20858784 10.1177/1087054710370935

[bib8] National Institute for Health and Clinical Excellence. Attention deficit hyperactivity disorder - Disagnosis and management of ADHD in children, young people and adults. 2008; Available at: http://publications.nice.org.uk/attention-deficit-hyperactivity-disorder-cg724/26/2012.

[bib9] Garfield CF, Dorsey ER, Zhu S, Huskamp HA, Conti R, Dusetzine SB et al. Trends in attention deficit hyperactivity disorder ambulatory diagnosis and medical treatment in the United States, 2000–2010. Acad Pediatr 2012; 12: 110–116. 22326727 10.1016/j.acap.2012.01.003 PMC3307907

[bib10] Brault MC, Lacourse E. Prevalence of prescribed attention-deficit hyperactivity disorder medications and diagnosis among Canadian preschoolers and school-age children: 1994-2007. Can J Psychiatry 2012; 57: 93–101. 22340149 10.1177/070674371205700206

[bib11] McCarthy S, Wilton L, Murray ML, Hodgkins P, Asherson P, Wong IC. The epidemiology of pharmacologically treated attention deficit hyperactivity disorder (ADHD) in children, adolescents and adults in UK primary care. BMC Pediatrics 2012; 12: 78. 22712630 10.1186/1471-2431-12-78 PMC3472167

[bib12] Wong IC, Murray ML, Camilleri-Novak D, Stephens P. Increased prescribing trends of paediatric psychotropic medications. Arch Dis Child 2004; 89: 1131–1132. 15557050 10.1136/adc.2004.050468 PMC1719746

[bib13] Garbe E, Mikolajczyk RT, Banaschewski T, Petermann U, Petermann F, Kraut AA et al. Drug treatment patterns of attention-deficit/hyperactivity disorder in children and adolescents in Germany: results from a large population-based cohort study. J Child Adolesc Psychopharmacol 2012; 22: 452–458. 23234588 10.1089/cap.2012.0022 PMC3523251

[bib14] Man KK, Ip P, Hsia Y, Chan EW, Chui CS, Lam MP et al. ADHD drug prescribing trend is increasing among school-aged children and adolescents. J Atten Disord 2014; pii:108705471453604 (e-pub ahead of print). 10.1177/1087054714536047 24994875

[bib15] Fazel M. Methylphenidate for ADHD. Br Med J 2015; 351: h5875. 26608974 10.1136/bmj.h5875

[bib16] Storebo OJ, Krogh HB, Ramstad E, Moreira-Maia CR, Holmskov M, Skoog M et al. Methylphenidate for attention-deficit/hyperactivity disorder in children and adolescents: cochrane systematic review with meta-analyses and trial sequential analyses of randomised clinical trials. Br Med J 2015; 351: h5203. 26608309 10.1136/bmj.h5203 PMC4659414

[bib17] Iversen L. Neurotransmitter transporters and their impact on the development of psychopharmacology. Br J Pharmacol 2006; 147: S82–S88. 16402124 10.1038/sj.bjp.0706428 PMC1760736

[bib18] Howes OD, Kambeitz J, Kim E, Stahl D, Slifstein M, Abi-Dargham A et al. The nature of dopamine dysfunction in schizophrenia and what this means for treatment. Arch Gen Psychiatry 2012; 69: 776–786. 22474070 10.1001/archgenpsychiatry.2012.169 PMC3730746

[bib19] Tost H, Alam T, Meyer-Lindenberg A. Dopamine and psychosis: theory, pathomechanisms and intermediate phenotypes. Neurosci Biobehav R 2010; 34: 689–700. 10.1016/j.neubiorev.2009.06.005 PMC2838993 19559045

[bib20] Vigilance and Intelligence Research Group. Yellow Card Scheme, 2015. Available at: http://www.mhra.gov.uk/drug-analysis-prints/drug-analysis-prints-a-z/index.htm (Accessed on 13 October 2015).

[bib21] Mosholder AD, Gelperin K, Hammad TA, Phelan K, Johann-Liang R. Hallucinations and other psychotic symptoms associated with the use of attention-deficit/hyperactivity disorder drugs in children. Pediatrics 2009; 123: 611–616. 19171629 10.1542/peds.2008-0185

[bib22] EMA. Summary of Product Characteristics. Available at: http://www.ema.europa.eu/docs/en_GB/document_library/Referrals_document/Methylphenidate_31/WC500011138.pdf (Accessed on 29 October 2013).

[bib23] EMEA. EMEA 2010 Priorities for Drug Safety Research: long-term effects in children and in young adults of methylphenidate in the treatment of attention deficit hyperactivity disorder (ADHD)2009. Available at: http://www.ema.europa.eu/docs/en_GB/document_library/Other/2010/03/WC500076318.pdf (Accessed on 19 September 2013).

[bib24] Shyu YC, Yuan SS, Lee SY, Yang CJ, Yang KC, Lee TL et al. Attention-deficit/hyperactivity disorder, methylphenidate use and the risk of developing schizophrenia spectrum disorders: A nationwide population-based study in Taiwan. Schizophr Res 2015; 168: 161–167. 26363968 10.1016/j.schres.2015.08.033

[bib25] Chan EW, Lau WCY, Leung WK, Mok MT, He Y, Tong TS et al. Prevention of dabigatran-related gastrointestinal bleeding with gastroprotective agents: a population-based study. Gastroenterology 2015; 149: 586–595.e3. 25960019 10.1053/j.gastro.2015.05.002

[bib26] Chui CSL, Man KKC, Cheng CL, Chan EW, Lau WCY, Cheng VC et al. An investigation of the potential association between retinal detachment and oral fluoroquinolones: a self-controlled case series study. J Antimicrob Chemother 2014; 69: 2563–2567. 24833754 10.1093/jac/dku145

[bib27] He Y, Chan EW, Man KK, Lau WC, Leung MK, Ho LM et al. Dosage effects of histamine-2 receptor antagonist on the primary prophylaxis of non-steroidal anti-inflammatory drug (NSAID)-associated peptic ulcers: a retrospective cohort study. Drug Saf 2014; 37: 711–721. 25096957 10.1007/s40264-014-0209-0

[bib28] Wong AYS, Root A, Douglas IJ, Chui CS, Chan EW, Ghebremichael-Weldeselassie Y et al. Cardiovascular outcomes associated with use of clarithromycin: population based study. Br Med J 2016; 352: h6926. 26768836 10.1136/bmj.h6926

[bib29] Chan EW, Lau WC, Siu CW, Lip GY, Leung MK, Anand S et al. Effect of suboptimal anticoagulation treatment with antiplatelet therapy and warfarin on clinical outcomes in patients with nonvalvular atrial fibrillation: a population-wide cohort study. Heart Rhythm 2016; 13: 1581–1588. 27033342 10.1016/j.hrthm.2016.03.049

[bib30] Man KK, Chan EW, Coghill D, Douglas I, Ip P, Leung LP et al. Methylphenidate and the risk of trauma. Pediatrics 2015; 135: 40–48. 25511122 10.1542/peds.2014-1738

[bib31] Chui CS, Chan EW, Wong AY, Root A, Douglas IJ, Wong IC. Association between oral fluoroquinolones and seizures: a self-controlled case series study. Neurology 2016; 86: 1708–1715. 27053716 10.1212/WNL.0000000000002633 PMC4854590

[bib32] Wong AYS, Wong ICK, Chui CS, Lee EH, Chang WC, Chen EY et al. Association between acute neuropsychiatric events and *Helicobacter pylori* therapy containing clarithromycin. JAMA Intern Med 2016; 176: 828–834. 27136661 10.1001/jamainternmed.2016.1586

[bib33] HAHO/ITDClinical Data Analysis & Reporting System (CDARS) User's Manual. In: Authority H (ed). 2nd edn, 2003, p. 3.

[bib34] Lai EC, Man KK, Chaiyakunapruk N, Cheng CL, Chien HC, Chui CS et al. Brief Report: databases in the Asia-Pacific region: the potential for a distributed network approach. Epidemiology 2015; 26: 815–820. 26133022 10.1097/EDE.0000000000000325

[bib35] Lao KS, Chui CS, Man KK, Lau WC, Chan EW, Wong IC. Medication safety research by observational study design. Int J Clin Pharm 2016; 38: 676–684. 27003827 10.1007/s11096-016-0285-6 PMC4909784

[bib36] Whitaker HJ, Farrington CP, Spiessens B, Musonda P. Tutorial in biostatistics: the self-controlled case series method. Stat Med 2006; 25: 1768–1797. 16220518 10.1002/sim.2302

[bib37] Oner O, Yilmaz ES, Karadag H, Vural M, Vural EH, Akbulat A et al. ADHD medication trends in Turkey: 2009-2013. J Atten Disord 2014 Feb 19. [Epub ahead of print]. 10.1177/1087054714523129 24554298

[bib38] Suhail K, Cochrane R. Seasonal variations in hospital admissions for affective disorders by gender and ethnicity. Soc Psychiatry Psychiatr Epidemiol 1998; 33: 211–217. 9604670 10.1007/s001270050045

[bib39] NICEAttention Deficit Hyperactivity Disorder: Pharmacological and Psychological Interventions in Children, Young People and Adults. The British Psychological Society and the Royal College of Psychiatrists: London, 2009.

[bib40] Musonda P, Farrington CP, Whitaker HJ. Sample sizes for self-controlled case series studies. Stat Med 2006; 25: 2618–2631. 16372391 10.1002/sim.2477

[bib41] Chakraborty K, Grover S. Methylphenidate-induced mania-like symptoms. Indian J Pharmacol 2011; 43: 80–81. 21455430 10.4103/0253-7613.75678 PMC3062130

[bib42] Lucas AR, Weiss M. Methylphenidate hallucinosis. JAMA 1971; 217: 1079–1081. 5109429

[bib43] MacKenzie LE, Abidi S, Fisher HL, Propper L, Morash-Conway J, Glover JM et al. Stimulant Medication and Psychotic Symptoms in Offspring of Parents With Mental Illness. Pediatrics 2016; 137: e20152486. 10.1542/peds.2015-2486 26719291

[bib44] Shibib S, Chalhoub N. Stimulant induced psychosis. Child Adolesc Mental Health 2009; 14: 20–23.

[bib45] Young JG. Methylphenidate-induced hallucinosis: case histories and possible mechanisms of action. J Dev Behav Pediatr 1981; 2: 35–38. 7263866

[bib46] Leung GM, Tin KY, O'Donnell O. Redistribution or horizontal equity in Hong Kong's mixed public-private health system: a policy conundrum. Health Econ 2009; 18: 37–54. 18264997 10.1002/hec.1342

[bib47] Chan CW, Lam C, Lau J, Lee T, Leung P, Liu S et al. Attention Deficit/Hyperactivity Disorder in Children 2007 Position Paper. The Hong Kong Society of Child Neurology & Developmental Paediatrics: Hong Kong, 2007.

