# Supplementary material for: Methylphenidate and the risk of psychotic disorders and hallucinations in children and adolescents in a large health system
Source: Transl Psychiatry. 2016 Nov 15;6(11):e956–. doi: 10.1038/tp.2016.216 (PMC5314128; doi:10.1038/tp.2016.216)
Supplement: Supplementary Table 1 [file tp2016216x1.docx]

**Supplementary Table 1 ICD-9-CM codes included**

| **Description** | ICD-9-CM^a^ |
| --- | --- |
| Psychotic disorder | 292.11, 292.12, 293.81, 293.82, 295.13, 295.14, 295.33, 295.34, 295.43, 295.44, 295.63, 295.64, 295.73, 295.74, 295.83, 295.84, 295.93, 295.94, 297.8, 297.9 |
| Other nonorganic psychoses | 298.0, 298.1, 298.3, 298.8, 298.9 |
| Hallucinations | 780.1 |

^a^ICD-9-CM=The International Classification of Diseases, Ninth Revision, Clinical Modification

**Supplementary Table 2 Distribution of events during MPH^a^ treatment and non-treatment periods**

| **Psychotic events** | MPH treatment period^b^ | (%) | Non-treatment period | (%) |
| --- | --- | --- | --- | --- |
| Psychotic disorder | 1 | 3.6 | 2 | 2.3 |
| Other nonorganic psychoses | 23 | 82.1 | 66 | 75.0 |
| Hallucinations | 4 | 14.3 | 20 | 22.7 |

^a^MPH=Methylphenidate ^b^Patients may have more than one condition on the event date
