# Supplementary figures and images for: Methylphenidate and the risk of psychotic disorders and hallucinations in children and adolescents in a large health system
Source: Transl Psychiatry. 2016 Nov 15;6(11):e956–. doi: 10.1038/tp.2016.216 (PMC5314128; doi:10.1038/tp.2016.216)

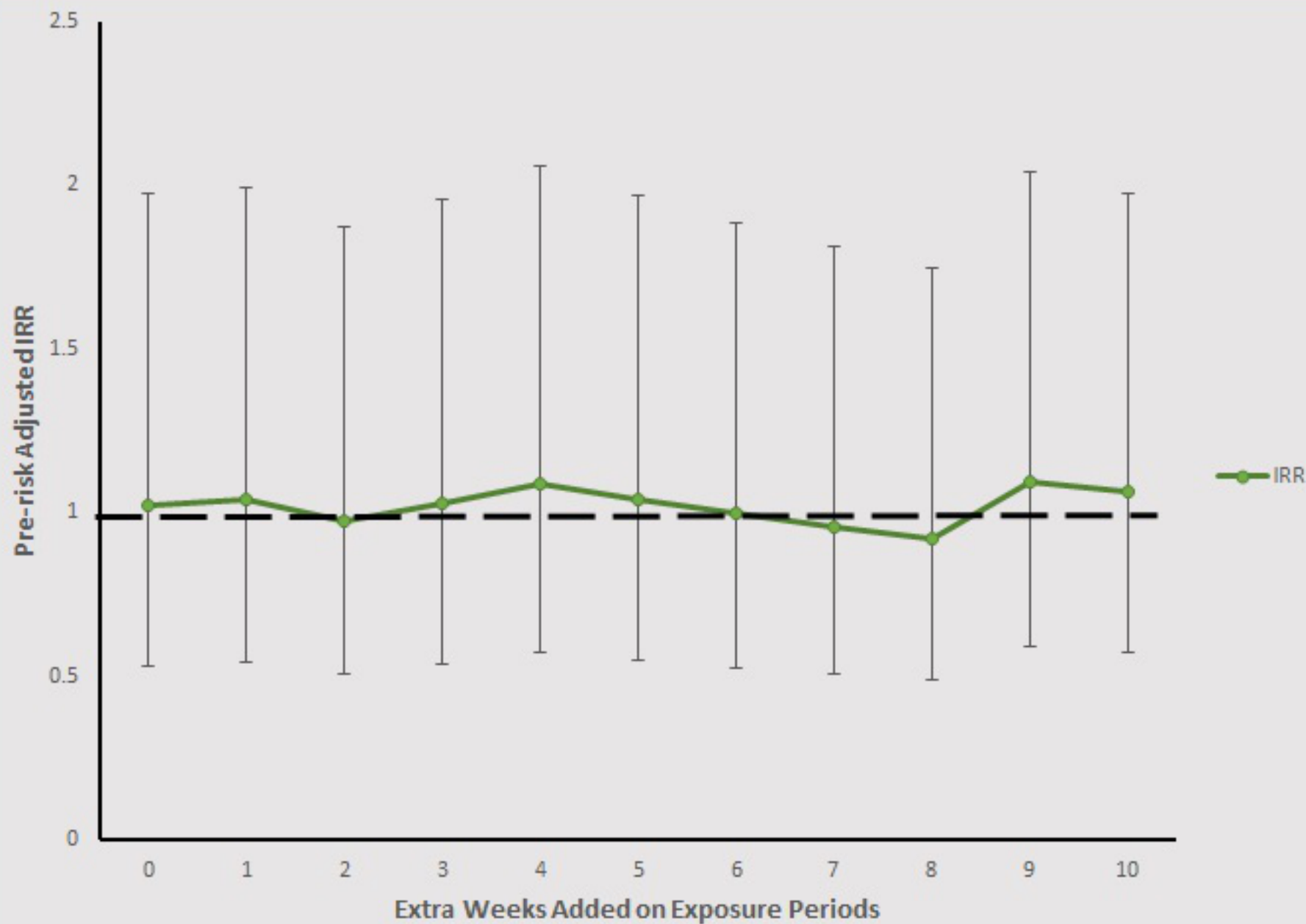

Supplement: Supplementary Figure 1 [file tp2016216x2.pdf]
